# Etherization: With Surgical Remarks

**Published:** 1848-03

**Authors:** 


### REVIEWS.

---

ART. IV.—*Etherization; with Surgical Remarks.* By JOHN C. WARREN, M.D., Emeritus Professor of Anatomy and Surgery in the University at Cambridge; Surgeon at Massachusetts General Hospital; Honorary Member of the Medical and Chirurgical Society of London; Corresponding Member of the Royal Academy of Medicine at Paris, and of the Academies of Naples, Florence, etc., etc. Boston: William D. Ticknor & Co. 12mo. pp. 100. 1848.

The author of this little work has long held a high rank among the first American surgeons, and his opinions are therefore certain to receive due consideration. It was at Boston that etherial inhalation, with a view to the annulling of pain in surgical operations, had its origin, and it is fit that Boston's most distinguished surgeon should give us the results of his experience with the new agent. We are glad of an opportunity of presenting to our readers the experience of so competent an observer, and proceed without further preface to notice the principal points of his exceedingly interesting tract. From the following passage it will be seen with what enthusiasm the veteran surgeon hails this wonderful discovery. He exclaims—

"A new era has opened to the operating surgeon! His visitations on the most delicate parts are performed, not only without the agonizing screams he has been accustomed to hear, but sometimes with a state of perfect insensibility, and occasionally even with the expression of pleasure on the part of the patient. Who could have imagined that drawing the knife over the delicate skin of the face might produce a sensation of unmixed delight! that the turning and twisting of instruments in the most sensitive bladder might be accompanied by a beautiful dream! that the contorting of anchylosed joints should co-exist with a celestial vision! If Ambrose Paré, and Louis, and Dessault, and Cheselden, and Hunter, and Cooper, could see what our eyes daily witness, how would they long to come among us, and perform their exploits once more! And with what fresh vigor does the living surgeon, who is ready to resign the scalpel, grasp it, and wish again to go through his career under the new auspices!"

The author, in the first few pages, gives a brief history of the progress of etherization, and, after remarking how slow this progress has been in this country compared with its rapid extension on the other side of the Atlantic, concludes this branch of his subject by stating, that he has assisted in operations where ether was employed in about two hundred cases, and has directed its use in a number of patients under his care where he was not present. The result of all this experience is stated in the following paragraph:

"We think it then fair to say, that the number of deaths from surgical operations after etherization, has not been greater than it would have been without. May it not be said even, that this number has been lessened by the exclusion of the shock from pain, which would have occurred, had it not been administered?"

It is a question, comparatively, of but little importance, how the ether makes its impression upon the system, whether through the medium of the nerves, or that of the blood-vessels. Dr. Warren believes that it is through the latter, and few, we presume, after a careful examination of the phenomena attending etherization, will come to any other conclusion. The fact, that the etherial odor is perceived in the blood a few minutes after the vapor is inhaled, must be held conclusive as to its imbibition.

It has been objected to etherization that asphyxia is sometimes induced by it, and that the blood is blackened, dissolved, and rendered slow to coagulate, in consequence of which hemorrhages are prone to occur. To this Dr. Warren replies—

"Such instances as these occurred, however, only in the early history of etherization, when the valved tube was employed to limit the entrance of atmospheric air, and produce a more exclusive inhalation of ether. As soon as the valve was removed, and especially after the introduction of the sponge, asphyxia ceased to occur, so that, in upwards of a hundred consecutive cases, there has not been more than a single instance, and this of short duration. There are no longer to be seen those violent struggles for breath, purple hue of the blood, or difficulty in the suppression of hemor rhage"

Dr. Warren is incredulous respecting the fatal action of ether in any of the cases in which death has been ascribed to it, at the same time that he acknowledges the potency of the agent, and would have it used with caution and judgment. He says—

"No instance of death in man from inhaling ether has occurred in our knowledge, or belief. For although there have been a number of cases reported as fatal, yet in these the connection between the cause and its supposed effect appears to be too imperfect to bear a close examination. Only one occurrence of this nature has happened here. A boy of very bad habits, whose arm was torn off by machinery, had an amputation at the shoulder-joint skilfully performed by Dr. Lewis on the day after the accident under etherization, and died in eight hours."

Respecting this issue Dr. Warren pertinently asks—

"Is there anything remarkable in the death of a boy, in the habit of using spirituous liquors freely, from a laceration of the arm near the shoulder, requiring an amputation at the joint?"

Dr. Warren does not believe, from all that he has seen, that etherization is at all likely to give rise to pulmonary affections, but, on the contrary, regards it as a good remedy in spasmodic asthma, and chronic bronchitis, in which it is a powerful expectorant.

It has been further objected to ether, that it may be employed in a criminal way; and the fact cannot be denied. That it has been thus abused evidence will be found on another page of this number; but that the abuse is not without its proper perils, the evil-disposed will be taught by the experience of the French dentist.

Some of the diseases in which Dr. Warren has witnessed the happy effects of ether, are colic, dysmenorrhea, and neuralgia; and aside from its agency in abolishing pain in all operations, he has found it eminently serviceable in strictures and dislocations, where spasmodic muscular contraction constitutes the chief embarrassment to the surgeon. He cites cases of stricture of the urethra, and of the œsophagus, in which he employed the remedy with the best effect.

But it is not in these circumstances alone that etherism promises to avail our race. There comes a time when all that the art of the physician can do, is to "smooth the stormy passage to the grave." As a euthanasial agent Dr. Warren thus speaks of it:

"Since the establishment of ethereal practice in surgical operations, its former utility in mitigating the agonies of death has led me to employ its influence in a more free and decided manner. And so far as the trials have extended, they serve to justify its use in a great number, and I hope I may say without enthusiasm, in the majority of instances. Should this prove, on a full trial, to be the fact, the value of the discovery will be greatly enhanced, since the number of those who are called on to suffer in the struggle between life and death, is greater than that of those who are compelled to submit to the pain of surgical operations.

"I am fully aware, that the agony in the dissolution of the bond between the bodily frame and its spiritual tenant is not so great as it is believed to be; for, having questioned a great number of persons passing through the last stage of earthly existence, whether they suffered pain, the answer has been almost uniformly in the negative; and on inquiring what sensation was experienced, the reply has been such as to lead me to consider it an undefinable sense of discomfort. The intellectual faculties appear to be so clouded and confused, that they are unable to take cognizance of the agitation which convulses the physical organization.

"There are, however, exceptional cases, in which there is great bodily suffering; and there is in all men an instinctive dread of the pains of death. If we find the means of preventing or relieving these pains, the great change may be viewed without horror, and even with tranquillity. He who would experience a real euthanasia should not, however, trust merely to the virtues of ether, but should also have settled his accounts with this world, and be well prepared to settle those of the future."

The following are the general conclusions at which Dr. Warren arrives on this interesting subject:

"1st. Inhalation of ether produces insensibility to pain.

"2d. Ethereal insensibility, judiciously effected, is not followed by any dangerous consequences.

"3d. Its administration is easy, and usually requires but a few minutes.

"4th. Individuals of all ages may be safely etherized.

"5th. Individuals of the same age are susceptible of the influence of ether in variable degrees.

"6th. Surgical operations may be done under the effect of ether, which could not be done without.

"7th Operations very short, and not very painful, especially those about the head and neck, are best done without ether.

"8th. The shock of the nervous system is greatly diminished by etherization.

"9th. The use of ether has increased the number of successful operations, by encouraging a resort to them at an earlier period of disease.

"10th. The use of the sponge is more safe and easy than that of any special apparatus.

"11th. A special apparatus is convenient for some peculiar cases.

"12th. The existence of chronic pulmonary disease rarely forms an objection to etherization.

"13th. Etherization may often be employed advantageously as a substitute for narcotics.

"14th. The employment of ether does not retard the healing of wounds, nor give them an unfavorable character.

"15th. The pains of death may often be relieved by etherization."

Nearly the whole of this volume was written before Chloroform was announced, and Dr. Warren devotes only a few pages to this subject; but the reader will not need to be told, that all that has been said of the virtues of ether is applicable to the substitute. Nay, there remains no longer a doubt, brief as has been the experience with chloroform, that this agent possesses many and decided advantages over ether, and is destined speedily to supersede it. From many trials with it in this city under our own observation, and among our professional friends, we are satisfied that its action is safe, agreeable, and unattended with danger. We doubt not since it was introduced here a few weeks ago, that a thousand persons have inhaled it in this city, and yet not a single accident has come to our knowledge, although many of the experimenters with it have been lads and boys in the streets. This is an abuse of the article, and it is to be hoped that the reprehensible practice will be suppressed, but thus far it has had the good effect pretty effectually to remove from the public mind all fear of the chloroform. Professor Simpson has lately published a case in the London Lancet strongly illustrative of the safety with which this vapor may be respired for even a long time. He kept a parturient female under its influence for *thirteen hours* consecutively, the labor being rendered tedious by the narrowness of the pelvic canal, and the child being at last delivered by the forceps. "If any permanently injurious influence were to be dreaded from the exhibition of chloroform," remarks a writer in the Medico-Chirurgical Review, "surely it would be manifested in such a case as this;" and yet, he adds, "we are assured by Professor Simpson that the child when born showed no other want of vital power than was fully accounted for by the long-continued pressure to which it had been subjected; and that the mother's recovery was remarkably rapid, the child also speedily becoming perfectly well."

Not a few physicians, distinguished for their talents and judgment, have kept aloof from the letheon excitement, and even regarded with distrust the warm enconiums bestowed upon it by their more enthusiastic brethren. Such, we think, must at length dismiss their prejudices, and if they could not adopt ether, at least give chloroform a trial. It is often a virtue to receive with allowance the testimony of discoverers in favor of new remedies, but to set one's face obstinately against the use of agents recommended as ether and chloroform are, is certainly *not* a virtue.

We do not hear of the use of these agents by many obstetrical practitioners in this country south of Boston, decided as is the evidence in their behalf by those who have made trial of them. In Scotland, we are told, the practice has been condemned by some on the ground that it contravenes the curse brought upon our first mother by her transgression in the garden. We wonder if it be possible that such an argument can have weight with any professional man? The objection strikes us as, in the last degree, absurd. To carry out the principle, we should set our faces against all the improvements introduced by science in the practice of midwifery, by which the pangs and perils of childbirth are diminished, and not only so, but must stoutly reject all the labor-saving expedients of our inventive age, for mitigating the toil of man, and thus lightening Adam's share of the burden.

We have not attempted to present a full analysis of the volume of Dr. Warren, purposing merely to give the results of his experience in etherization; and leave the reader to derive from the work itself the many valuable suggestions which it contains on various surgical subjects.

